# Suggestions for the Construction of Artificial Crowns and Bridges

**Published:** 1884-12

**Authors:** J. H. Spaulding

**Affiliations:** Hanover, Germany


### SUGGESTIONS FOR THE CONSTRUCTION OF ARTIFICIAL CROWNS AND BRIDGES.

BY DR. J. H. SPAULDING, HANOVER, GERMANY.

READ BEFORE THE AMERICAN DENTAL SOCIETY OF EUROPE, AT ITS MEETING AT VEVEY, SWITZERLAND, 1884.

The discussion of the merits and claims of artificial crown and bridge work is likely to have somewhat of a revolutionizing effect, in both the operative and mechanical branches of dentistry. It will eventuate, as have all other marked departures from the ordinary routine teachings in dentistry, in two parties; there will be some who will hail this practice as a method to be urged at all times and under all circumstances and conditions, and who will recognize no difficulty too great to be "bridged over" by porcelain and gold. It is natural that it should be so. There will be enthusiasts in every new field, and especially in any department which has for its foundation principles of value, and capable of being used for great good and advantage. In every such field enthusiasts are apt to go too far, and to assume that a principle of good cannot become one of positive injury if improperly or injudiciously applied. Others will occupy the other extreme, and condemn, ofttimes without a hearing, any departure from the known and tried methods with which they are familiar.

Were it not for these two extreme classes which serve to keep each other in check, we might not be sufficiently attentive to look for the medium which serves the greatest good to the greatest number. For the claims and pretentions of the artificial crown work I have the highest respect and admiration, because I believe that through the methods prescribed we may be able to save many teeth which have heretofore been sacrificed because we did not know exactly how to deal with them. Dr. J. L. Williams, of Hartford, Conn., has written excellently upon this subject. I shall not rehearse what he has said, but will only suggest the methods of making the gold, and gold-and-porcelain crowns for molars and bicuspids, which I am using. My manner of making the incisor and cuspid crowns is the same as his, and is, no doubt, familiar to you all.

After preparing the root so that its diameter is nowhere greater than just at the free margin of the gum, I take a narrow strip of sheet lead or tin, of sufficient thickness to bend easily and remain in any position, and adjust it to the root just where I want my gold crown to fit most closely. Pressing with suitable burnishers into any irregularities, and bringing the ends squarely together, I have the exact length of the gold strip which must encircle the root. Using for this 22 k. gold of about 32 English standard gauge, I now cut it off and bend it with pliers to fit the irregularities, as I did the lead, and bring the ends squarely together and solder. All this takes perhaps thirty minutes. I now place my gold band upon the root, pushing it up as far as I intend it to go when finished, and take an impression with this ring in position, remove the impression and replace the ring in the proper position therein, and fill with plaster. I now have a model of the case with the gold ring upon the root I am crowning. I take also an impression of the opposite jaw to secure the articulation. With the models of both jaws, and the proper articulation, I can fit and shape my crown as I need, using strong resin wax to build up for occlusion with the opposing teeth.

When the proper shape is secured, take 24 k. gold, rolled very thin, say No. 38 or 40 English standard, and burnish over the outside of the wax, bringing the edges down outside the narrow gold ring. I now invest in plaster, and remove from the model, taking out all the wax from the inside, and flow solder over the entire surface of the inside of the crown. This stiffens the 24 k. gold, and makes it sufficiently thick to resist wear in use.

With a little polishing it is ready to put in position, and is of better shape than it is possible to secure in any other manner, save by swaging with dies, and all is done easily and quickly. If it is desirable to have a porcelain face and grinding surface, as is sometimes the case in bicuspids and first molars, I make my ring as before, only using a gold strip as wide as the full length of the desired crown. I place this ring upon the root and take my impressions as above, making my models and securing the proper articulation. I now cut away from the buccal wall of the gold ring down to within a line of the gum margin, and select the proper porcelain molar or bicuspid, fit it into the ring, grinding the porcelain, or bending the gold as may be necessary, until the fit is secured. I next place my ring in position in the mouth, driving it well up, and dry off the end of the root, using the rubber dam if possible, and fill the band with Poulson's cement, and press my porcelain face into position. Removing the excess of cement, and allowing, of course, a few minutes for it to harden, my crown is finished. In cases where necessary, I always place a headed pin in the root canal, either with cement or amalgam, before placing the crown in position; the head of the pin is allowed to stick out, to be grasped by the cement with which the crown is filled.

No objection which has been urged against the "bridge" by its opponents can have any application to the crowns as described above, or in the article of Dr. Williams. The root, if properly treated, is as safe as any tooth which has lost its vitality, and is thoroughly protected from further decay; and, if well done upon a reasonably good root, it will take care of itself for many years. There is also a large class of teeth which are wasted by abrasion and some forms of dry decay almost to the point of pulp exposure, and which are not amenable to treatment that will conserve their vitality by any method of filling, and which can only be securely protected by capping.

For the principles upon which the "bridge" is constructed, and upon which it bases all its claims to public favor, I also have high respect, and I believe that under some conditions—many perhaps—the bridge, properly made, will prove serviceable, comfortable, and as cleanly as the natural teeth in the same position.

It is to the almost unlimited pretensions of the bridge-work enthusiasts that I object. They only limit themselves to "four roots in the proper position," upon which they engage to construct an entire denture, without any plate whatever.

This looks inviting upon paper, and encouraging to those unfortunates who are obliged to wear artificial teeth, and proves, no doubt, a good advertising medium, but I doubt very much the propriety of such extended bridges, and believe they will prove uncleanly, of comparatively short duration, easily broken, and with difficulty repaired, as well as very expensive.

To meet the requirements in cases where the permanent bridge is not practicable, I beg to suggest some methods which can be utilized with much comfort and satisfaction to the patient. A case commonly presenting is one where all the molars and second bicuspids of the lower jaw are gone, and is one for which it is always extremely difficult, and sometimes almost impossible, to make a plate that will set easily and remain in place, entirely dependent upon suction. If, as is frequently the case, there is decay in the first bicuspids, they are to be cut off and prepared as for the single crown. Take a sharp impression of these roots, and make a gold cap to fit the exposed end of each; make a hollow cylinder of gold, and a pin to fit the cylinder. Now, after enlarging the nerve canals, place the caps in position and punch holes through the top to correspond with the nerve canal opening, and place the cylinders, cut to the proper length, through the opening in the cap into the canals; fasten with resin wax, remove and solder. This done, place permanently in position upon the roots with cement (using, preferably, Poulson's or Rostaing's). Next place in the metal canals the pins already made to fit them, the ends projecting, and take an impression as for any ordinary plate. The pins will come away with the impression, and show exactly the position and length of the canals. Now place *upon these pins* cylinders of any metal most convenient (brass, lead, tin), and fill the impression with plaster for the model; thus you have in the model the exact counterparts of the metal canals in the mouth. You now place the pins in these canals as you did in the canals in the roots before taking the impression, allowing the ends to project as before, and proceed to make the plate as for any ordinary case. It is well to flatten, or roughen, or head the ends of the pins projecting from the canals in the model, so that the rubber—if rubber is used—will more firmly grasp and hold them. When your piece is finished it will set easily and steadily in the mouth, and is not readily moved about by the tongue or muscles, but it can be easily removed for cleansing, and the amount of plate material you need is reduced to the minimum. Another advantage is that the plate need not touch any of the natural teeth remaining in the mouth, thus preventing decay, which plates so often cause. Another case: molars and bicuspids gone, except wisdom teeth, which are badly decayed. Grind down the crowns of these teeth, shaping them properly, and make gold caps for both, and on the top of each solder a short, square pin, and when finished place in position upon the roots with cement. Now take an impression and make up your denture in the usual way, allowing the material of which the plate is composed to extend over the gold crowns, fitting closely around the pins described above. The caps over the wisdom teeth serve a double purpose; that of preserving these teeth and affording a support or fastening for the denture.

Another case shows the lower teeth all gone, except cuspids, which are either sound, or have good roots and in good condition. Extract all roots except cuspids; cut these off and proceed as before to make the gold caps with canals and pins, and place in position with cement, continuing exactly as described for the first case. When complete you have a lower denture entire, which stays where it is put in the mouth in spite of stubborn muscles and tongue, with the roots thoroughly protected and safe to support the denture for many years. If the pins should become a trifle loose by wear, they can be split and opened a little so as to spring into the canals, thus holding more firmly. The cases I have cited are only those of the lower jaw, but the same principles apply with equal advantage to any such case on the upper, or any modification of these conditions upon either jaw. These suggestions may not be new to many of the members of your society, but they will, I am sure, be of some value to many operators in America, where the custom of extracting all teeth and roots preparatory to inserting the artificial denture has more generally obtained than in Germany. That such a practice is a thing of the past there are already hopeful signs, and the employment of the methods suggested herein is better adapted to the purse and skill of patients and operators in the ordinary walks of the profession, than the very expensive bridges requiring a high degree of skill in construction, while possessing some advantages over the latter.

